# Blinding and sham control methods in trials of physical, psychological, and self-management interventions for pain (article II): a meta-analysis relating methods to trial results

**DOI:** 10.1097/j.pain.0000000000002730

**Published:** 2022-07-11

**Authors:** David Hohenschurz-Schmidt, Jerry Draper-Rodi, Lene Vase, Whitney Scott, Alison McGregor, Nadia Soliman, Andrew MacMillan, Axel Olivier, Cybill Ann Cherian, Daniel Corcoran, Hilary Abbey, Sascha Freigang, Jessica Chan, Jules Phalip, Lea Nørgaard Sørensen, Maite Delafin, Margarida Baptista, Naomi R. Medforth, Nuria Ruffini, Stephanie Skøtt Andresen, Sylvain Ytier, Dorota Ali, Harriet Hobday, Anak Agung Ngurah Agung Adhiyoga Santosa, Jan Vollert, Andrew S.C. Rice

**Affiliations:** a Pain Research, Department Surgery & Cancer, Faculty of Medicine, Imperial College, London, United Kingdom; b Research Centre, University College of Osteopathy, London, United Kingdom; c Department of Psychology and Behavioural Sciences, Section for Psychology and Neuroscience, Aarhus University, Aarhus, Denmark; d Health Psychology Section, Department of Psychology, Institute of Psychiatry, Psychology and Neuroscience, King's College London, London, United Kingdom; e INPUT Pain Management Unit, Guy's and St Thomas' NHS Foundation Trust, London, United Kingdom; f Human Performance Group, Department Surgery & Cancer, Faculty of Medicine, Imperial College, London, United Kingdom; g Faculty of Medicine, Imperial College London, London, United Kingdom; h Chemical Engineering Department, Khalifa University, Abu Dhabi, United Arab Emirates; i; j Department of Neurosurgery, Medical University Graz, Graz, Austria; k Résidence les Estrangers, La Bourboule, France; l Department of Occupational Medicine, Danish Ramazzini Centre, Aarhus University Hospital, Aarhus, Denmark; m The Penn Clinic, Hertfordshire, Hatfield, United Kingdom; n Wolfson Centre for Age Related Diseases, Department of Psychology, Institute of Psychiatry, Psychology and Neuroscience, King's College London, London, United Kingdom; o London, United Kingdom; p National Centre Germany, Foundation C.O.M.E. Collaboration, Berlin, Germany; q Department of Psychology and Behavioural Sciences, Aarhus University, Aarhus, Denmark; r Vevey, Switzerland; s Genetic and Developmental Psychiatry Centre, Institute of Psychiatry, Psychology and Neuroscience, King's College London, London, United Kingdom; t Institute of Psychiatry, Psychology and Neuroscience, King's College London, London, United Kingdom; u Denpasar, Indonesia; v Division of Neurological Pain Research and Therapy, Department of Neurology, University Hospital of Schleswig-Holstein, Campus Kiel, Germany; w Department of Anaesthesiology, Intensive Care and Pain Medicine, University Hospital Muenster, Münster,Germany; x Neurophysiology, Mannheim Center of Translational Neuroscience (MCTN), Medical Faculty Mannheim, Heidelberg University, Heidelberg, Germany

**Keywords:** Randomized controlled trials, Placebos, Placebo effect, Control groups, Systematic review, Meta-analysis, Physical therapy modalities, Rehabilitation, Psychotherapy

## Abstract

Sham interventions in randomized clinical trials (RCTs) of physical, psychological, and self-management (PPS) therapies for pain are highly variable in design and believed to contribute to poor internal validity. However, it has not been formally tested whether the extent to which sham controls resemble the treatment under investigation consistently affects trial outcomes, such as effect sizes, differential attrition, participant expectancy, and blinding effectiveness. Placebo- or sham-controlled RCTs of PPS interventions of clinical pain populations were searched in 12 databases. The similarity of control interventions to the experimental treatment was rated across 25 features. Meta-regression analyses assessed putative links between employed control interventions, observed effect sizes in pain-related outcomes, attrition, and blinding success. The sample included 198 unique control interventions, dominated by manual therapy and chronic musculoskeletal pain research. Meta-analyses indicated small-to-moderate benefits of active treatments over control interventions, across subgroups of manual therapies, exercise, and rehabilitation, and psychological intervention trials. Multiple meta-regression modelling demonstrated that similarity between sham control and tested interventions predicted variability in pain-related outcomes, attrition, and blinding effectiveness. Influential variables were differences relating to the extent of intervention exposure, participant experience, and treatment environments. The results support the supposed link between blinding methods and effect sizes, based on a large and systematically sourced overview of methods. However, challenges to effective blinding are complex and often difficult to discern from trial reports. Nonetheless, these insights have the potential to change trial design, conduct, and reporting and will inform guideline development.

## Abstract 2

Supplemental Digital Content is Available in the Text.

## 1. Introduction

Clinical trials are believed to show larger effects when they are not blinded.^45,57^ Blinding or masking refers to preventing trial participants from knowing which of the available treatments they receive, so that such knowledge does not affect their expectation of treatment benefit and thus bias trial results. Expectancies are widely assumed to mediate health benefits due to contextual factors: the placebo effect. Further placebo mechanisms include learning and conditioning, where various symptom-modifying neurobiological pathways are activated and which are dependent on contextual factors of a treatment. Related placebo contributors are the therapeutic interaction and the treatment's meaning to patients.^7,26,48^

In clinical trials, “placebo” or “sham” control interventions are used for blinding and to control for the psychosocial context of the treatment, spontaneous remission, and other confounding factors.^48,76^ In principle, this can be achieved through a therapeutic ritual that does not include features of the experimental treatment believed to produce the treatment effects and subject to study in a given trial. The concept of placebo controls is best illustrated by drug studies, where identical pills can be used, not containing the pharmacologically active agent but otherwise allowing for the same doctor interactions and rituals of pill taking.^76^ However, nonpharmacological therapies are often more complex in their procedures and more reliant on patient–provider interactions than drug therapies. Furthermore, the distinction between specific and contextual treatment components can be highly challenging and controversial.^23,61,77^ Consequently, blinding is more difficult in trials of nonpharmacological treatments.^16^ In nonpharmacological studies, such specifically designed control interventions are usually called “sham” or “attention controls,” despite slight differences in meaning.

For complex intervention studies, inert pills are not considered adequate controls, mainly because less elaborate placebos produce less pronounced placebo effects.^46,54,89^ Efficacy trials of complex interventions require complex control interventions, matching some or most features of the intervention. For example, sham ultrasound is often used in manual therapy trials, which is a largely dissimilar complex control treatment in this context. While low-similarity shams are easy to design and have, on occasion, been shown to effectively blind participants to group allocation,^8^ they may have a higher risk of unblinding and are unable to control for influential but unknown contextual factors. Importantly, trials that use dissimilar controls are believed to overestimate treatment efficacy.^5^ Furthermore, the placebo response is highly variable,^78,83^ and it is often unclear which aspects of the psychosocial treatment context influence the individual trial and to what extent, making it desirable to fully replicate the experimental treatment's context.

Therefore, a high degree of similarity between control and experimental interventions is commonly recommended for efficacy trials,^3,5,18,34,39,52,64,75^ but quantitative data to substantiate such recommendations are sparse. Some authors have used the concepts of “indistinguishability,” “sham fidelity,” and “structural equivalence” to denote this similarity.^5,52,64^ Despite “indistinguishability” being commonly recommended, it has not been systematically assessed which aspects in the resemblance between experimental and control interventions predict differences in trial outcomes. Such knowledge would enable researchers to prioritise and target efforts during the development of novel control interventions for efficacy trials, acknowledging the practical constraints of clinical trials. The present meta-analysis draws on previous work to define numerous features across which control and treatment interventions may be compared,^3,5,14,17,18,21,24,34,39,45,64,68,73,75^ but it refrains from a dichotomous distinction into “similar” and “dissimilar.”

There are currently no guidelines for dedicated control interventions in efficacy or mechanistic trials of physical, psychological, and self-management therapies (PPS) for pain. Such guidelines can only be sensibly developed based on improved insights regarding the effect of design decisions on trial feasibility, participant blinding, and outcomes. As such, the question of whether similarity between test and control interventions matters is of primary importance. Also, with blinding effectiveness rarely reported in trial publications,^41^ the retrospective assessment of control intervention quality would be facilitated by establishing quality standards. To advance this field, a systematic review of sham-controlled RCTs of PPS interventions was conducted. In a linked publication, we have reported the results of similarity assessments between control and experimental treatments, and information on additional blinding methods, control intervention development processes, blinding assessments, and reporting guideline compliance.^41^ Here, we compare the control and tested interventions across multiple features and test whether different levels of similarity between interventions predict differences in trial results, including pain-related outcomes, blinding effectiveness, and differential attrition.

## 2. Methods

Reporting of this systematic review follows the PRISMA 2020 statement.^62^ Further methodological detail is reported in a parallel article^41^ where the included trial methods were described in detail, including discussion of similarity features between interventions and differences between therapy groups. In short, the following methods were used for the systematic search, record screening, and data extraction.

### 2.1. Protocol and registration

A protocol was registered with the International Prospective Register of Systematic Reviews (PROSPERO) (registration ID: CRD42020206590). This publication reports the second part of the review, including outcome data and the meta-analysis. Protocol deviations occurred in relation to the employed meta-analysis methods as specified below.

### 2.2. Eligibility criteria

We included randomized controlled trials of PPS interventions for adults living with pain. Studies had to report at least one pain-related outcome measure. PPS interventions included all forms of manual and physical therapy; exercise and rehabilitation therapy; conversation-based and psychological therapies; body–mind, spiritual, religious, and other nonmaterial healing practices; web-based treatments; relaxation; and educational interventions (the latter 2 were classified as “self-management” here). We excluded drug studies, surgery, nutrition and infusions, device-based treatments, acupuncture and dry needling, and any other treatment based on meridian or reflex point considerations because these provide different challenges and opportunities for control intervention design than the group of nonpharmacological interventions studied here.^6,18^ To be eligible for this review, trials had to use a sham control intervention (or “attention” or “placebo control”). Pilot or feasibility studies were only included if they specifically assessed features of the control intervention in a pain population, irrespective of employed outcome measures (henceforth called validation studies). This review systematically assessed studies published from 2008 onwards.^15^

### 2.3. Data sources

The following databases were searched from January 2008 to 24 November 2021. MEDLINE, EMBASE, PsychInfo, the Cochrane Database of Systematic Reviews, the Cochrane Central Register of Controlled Trials (CENTRAL), National Institutes of Health Clinicaltrials.gov, AMED (Allied and Complementary Medicine), CINAHL (nursing and allied health), the Physiotherapy evidence database (pedro.org.au), ostmed.dr (ostmed-dr.oclc.org), osteopathic research web (osteopathic-research.com), and the index to chiropractic literature (chiroindex.org). The year 2008 was used as a cutoff because the first reporting guideline for nonpharmacological trials was published in that year.^15^

### 2.4. Search strategy

The search strategy was built around the following keywords and is provided in full for each database as supplement (spreadsheet, Supplemental Digital Content 1, available at http://links.lww.com/PAIN/B675). Aligning with the PICOS framework, this review's target population, interventions, control conditions, outcomes, and study designs are encompassed by the following:

Pain OR painful conditions AND Physical, Psychological, Self-management therapies (specific therapy and technique names) AND placebo control OR sham control OR attention control AND controlled clinical trials. Limit: 2008-present. Pain-related outcome measures were not searched for specifically.

### 2.5. Study selection

Eligibility screening was performed in duplicate by 2 independent reviewers drawn from a pool of specifically trained research contributors. Disagreements were resolved by a third independent reviewer. Screening was first performed based on study title and abstract and next based on the full text.

Instead of excluding smaller trials over risk of bias concerns,^30,31,37,84,85^ this review provides a descriptive overview of existing, otherwise eligible, trials of less than 20 participants per arm at randomization, allowing for the potential identification of novel or promising alternative methods of placebo controlling. Furthermore, we included these smaller studies in sensitivity analyses of the meta-regression modelling. Thus, and in extension of our initial protocol, outcome data were extracted, and risk-of-bias assessments performed for these trials.

### 2.6. Data extraction

Data extraction also required 2 independent reviewers, with discrepancies resolved through discussion or by a third independent reviewer. In trials with more than one sham control group, data for all sham control groups were extracted and treated independently in the analysis. Publications reporting multiple sham controls were assessed independently for each pair of intervention and control intervention, with data from an active intervention arm used twice for comparisons with control interventions if required (sample size of the respective group was halved to account for duplicate inclusion). Where a single sham group acted as comparator for multiple active interventions, data were extracted from the active intervention that most resembled the control intervention.

The domains of data extraction were bibliographic information, general study design, trial reporting, sham control and blinding methods, trial result (including attrition and blinding effectiveness), and risk of bias using the Cochrane Risk of Bias tool 2 (RoB 2).^71^ Resemblance between sham and experimental treatments was rated for 25 features.

Pain-related outcome measures were extracted for sham control and active intervention groups at baseline and earliest follow-up after treatment. Pain-related outcomes can be unidimensional (usually pain intensity rating scales) or multidimensional (eg, questionnaires assessing disease-related function, pain interference or quality of life).^74^ In the absence of research on whether these are differentially susceptible to placebo effects, we strove to extract both a unidimensional and a multidimensional outcome measure per study. Specifically, data were sampled for the declared primary pain-related outcome, irrespective of its nature. Where available, another pain-related outcome of the opposite dimensionality was extracted. Where authors did not declare a primary outcome measure, data for a unidimensional and a multidimensional measure were extracted if available. Direction of effect was considered. Where necessary, data were extracted from figures using the Adobe Reader measurement tool. Authors were contacted through email if data were missing that were required for the calculation of effect sizes or if data appeared erroneous. For cross-over designs, only results from the first phase were extracted.

### 2.7. Data analysis

#### 2.7.1. Descriptives and subgroups

A descriptive overview of blinding methods used in the field of PPS interventions for pain is provided in a parallel publication^41^ and the Supplemental Digital Content 2 (available at http://links.lww.com/PAIN/B676), including a basic description of placebo controlled interventions, their development and reported rationale, the similarity between control and active interventions, compliance with relevant reporting guidelines, and reports of blinding effectiveness.

Studies were subgrouped into large and small trials or by therapy type where appropriate. Trials of less than 20 participants per arm at randomization and placebo control validation studies without pain-related outcome measures were not used for primary meta-analyses but included in sensitivity analyses. Trials that could not be pooled were only analysed descriptively.^41^ Criteria for nonpooling were pain or disability that was not expected to improve in a comparable manner over the course of the study (cancer- and pregnancy-related pain) and studies with patients who had no pain or pharmaceutically reduced pain at outset (pain interventions during or immediately after surgery). Outliers were checked for errors in data reporting or entry and removed if errors in the primary data were suspected or if between-group standardised mean changes deviated from the group mean by more than 2.5 standard deviations.

#### 2.7.2. Meta-analysis: placebo responses and treatment effects

For all studies with more than 20 participants per arm, we synthesised outcome data and present a risk of bias (RoB) assessment.^71^ For each included control group, placebo responses were calculated as standardised mean changes (SMC) from baseline to first follow-up after the end of the treatment period, for both a uni- and a multidimensional pain-related outcome measure, where available. To calculate SMCs, a change score was divided by the pooled standard deviations.

Between-group differences were calculated as the difference in SMCs between active and control groups for the same measures and timepoints. Meta-analyses of between-group differences were performed per therapy subgroup and for uni- and multidimensional measures, separately. For each group of therapies, summary effects were calculated using random effects models weighted by the inverse of the variance and plotted using RevMan 5 software.^72^ The heterogeneity of overall effects was estimated using Tau^2^ (*T*^2^) and I^2^ statistics and tested for significance using Z statistics.^13^ Data are presented in forest plots, also illustrating study-specific risk of bias.

#### 2.7.3. Meta-analysis: similarity ratings

“Similarity ratings” were calculated by converting the evaluation of how similar individual features were between active and sham control interventions into numerical scores. Specifically, clear “Yes” (similar) and “No” (dissimilar) evaluations were rated as 2 and -2, respectively. “Probably Yes” and “Probably No” were awarded 1 and −1 points, and 0 points were given for items that could not be rated because of insufficient information. Nonapplicable items were not rated. Results of these ratings are presented in the parallel publication^41^ and as supplement to this article (Supplemental Digital Content 3, table, available at http://links.lww.com/PAIN/B677). These data were used as covariates for meta-regression in this review.

#### 2.7.4. Meta-analysis: blinding effectiveness, blinding indices, and treatment expectations

During data extraction, we identified all studies that provided an indication as to the effectiveness of the employed blinding methods. Where group guesses were reported in a manner that allowed for the calculation of the Bang blinding index (BI), the index was calculated for active and control groups individually^4^ and a ratio calculated as Hedge g for each comparison between active and sham control groups.^25^ Descriptive results are again provided as a supplemental table (Supplemental Digital Content 4, available at http://links.lww.com/PAIN/B678). The effect size of Hedge g was estimated irrespectively of the interventions studied, using meta-analysis methods as above.

In addition, we identified trials that reported measuring participant expectancy or related concepts (treatment credibility and satisfaction). On the suggestion of a reviewer and not specified in the original protocol, we examined these reports for the possibility of data pooling and meta-analysis of expectancy measures. For meta-analysis and regression with similarity ratings, reported expectancy data had to be (1) clearly attributable to expectation of treatment benefit only (ie, not presented as a compound measure with questions on treatment credibility or evaluated as treatment satisfaction), (2) sampled after at least one exposure to the test or control intervention but not after the final of multiple treatment sessions (to avoid confounding with treatment satisfaction), and (3) reported in full and per trial group.

#### 2.7.5. Multiple meta-regression analysis: the role of similarity between intervention and sham controls in predicting trial outcomes or blinding effectiveness

Based on interim feedback from subject experts, we deviated from the preregistered protocol to perform a more rigorous meta-regression analysis instead of simple correlation testing. Specifically, to assess the potential relationship between the trial results and how (dis)similar sham and active interventions were, for each individual therapy subgroup, meta-regression analyses were performed using methods-of-moments random effects models.^13,86^ The SMC between sham control and active intervention groups was used as the dependent variable, and models were weighted by the inverse of their variances. Models were built for each subgroup individually, identifying potential predictive variables from the pool of similarity ratings. This was accomplished based on nonparametric correlation analyses between all ratings for each subgroup to identify a selection of variables with little interdependence between each other and then further refined by iterative adjustment of the model until a model of supposed best fit was found. Put simply, we tested if similarity ratings could account for some of the differences in pain-related outcomes between trials. In particular, this method examined differences that were not likely because of the “true” difference in treatment efficacy but because of other factors, for example, control methods.

Meta-regression modelling was also performed for a subgroup of studies for which the Bang blinding index could be calculated, testing whether similarity features could predict variance in the studies' blinding success as well as testing whether the blinding index could predict the variance in studies' effect sizes. These analyses were irrespective of the type of therapy tested in the trials. Similarly, 2 meta-regression models were computed to examine whether differences in attrition between studies predicted trial outcomes and, furthermore, whether the degree of similarity between active and sham interventions predicted the degree of differential attrition. The results of meta-regression models are presented per therapy group below. Primary analyses were performed with large studies only (20 or more participants per group), and sensitivity was tested using the complete data set and/or excluding studies that did not formally qualify as outliers (ie, were included in the meta-analysis and primary modelling) but whose confidence intervals did not overlap with those of the aggregate effect.

We initially planned to use a trial-level average of similarity ratings for meta-regression analyses but decided to use ratings for individual items only, given concerns about the validity of a compound score. Notably, many of the individual items' ratings were intercorrelated so that an overall score would have been biased. Also, an equal weighting of all items as part of an average was seen as an undue assumption. We do, however, explore the average of all similarity ratings as part of our descriptive analysis (reported separately^41^).

## 3. Results

### 3.1. Sample description

**Figure 1.**
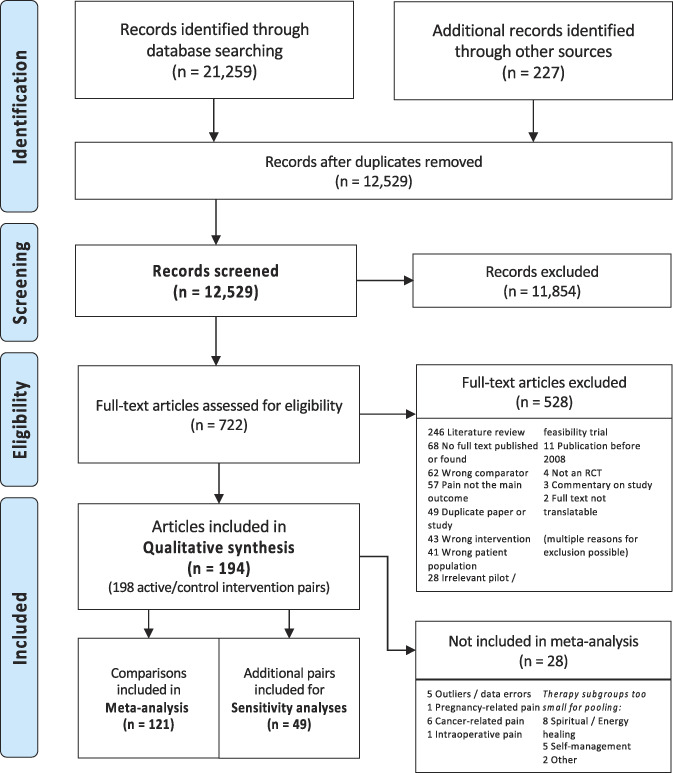
PRISMA flow diagram of the systematic search and selection process. Complete search strings per database and a list of all studies excluded at the full-text screening stage are provided in the Supplementary Digital Content file 1, available at http://links.lww.com/PAIN/B675.

Included trials' characteristics are illustrated in Table 1. Figure 1 shows the study selection process. We reviewed 194 publications (plus protocols where available), extracting data for 197 unique sham interventions and 198 comparisons between sham and experimental interventions. Manual therapy trials were most common, and there were multiple psychological and rehabilitation trials. Mostly, patients with musculoskeletal pain were recruited. While sham control interventions were not always well described, we were able to classify a range of employed methods, including control interventions that were clearly modelled based on the active treatment under investigation and such that were very dissimilar. Further describing levels of similarity between control and experimental interventions, we identified features for which similarity was frequently given, such as the amount and frequency of treatments. For other aspects, similarity was more variable, often also depending on the category of intervention studied. The first part of this results section will describe the entire sample, highlighting the subset of large trials eligible for primary meta-analyses, whereas the second part presents the meta-analysis of eligible studies only (Table 1). A table with each study's details is provided as supplement (Supplemental Digital Content 2, available at http://links.lww.com/PAIN/B676).

**Table 1:** Sample overview.

Therapy types	Total sample		Large trials		Small trials	
	n	%	n	%	n	%
Manual therapy with spinal manipulation	48	24.2	36	25.0	12	22.2
Craniosacral therapy and gentle myofascial release	22	11.1	16	11.1	6	11.1
Other manual therapy	64	32.3	40	27.8	24	44.4
Rehabilitation/physiotherapy	22	11.1	16	11.1	6	11.1
Self-management	5	2.5	4	2.7	1	1.9
Cognitive behavioural and other psychotherapy	27	13.6	26	18.1	1	1.9
Spiritual/energetic/esoteric healing	8	4.0	5	3.5	3	5.6
Other	2	1.0	1	0.7	1	1.9
Intervention complexity*						
Simple	112	56.6	73	50.7	39	72.2
Complex	82	41.4	71	49.3	11	20.4
Pain descriptor						
Musculoskeletal pain	121	61.1	88	61.1	33	61.1
Headaches and orofacial pain	22	11.1	15	10.4	7	13.0
Diffuse chronic pain	18	9.1	16	11.1	2	3.7
Injury-related and medical intervention-related pain	19	9.6	8	5.6	11	20.4
Cancer-related pain	6	3.0	6	4.2	0	0.0
Visceral pain	5	2.5	4	2.8	1	1.9
Neuropathic pain	5	2.5	5	3.5	0	0.0
Pregnancy-related pain	1	0.5	1	0.7	0	0.0
Preregistered trial protocol available						
Preregistered	114	57.9	90	62.9	24	44.4
Total included	Total sample		Large trials		Small trials	
	198		144		54	

The types of therapies, intervention complexity, and pain population are provided for the entire sample and per group. “Large” trials had 20 or more participants per arm, and their pain-related outcome data were used for meta-analyses. Special cases (large trials): In one trial, data from the active intervention group was used twice to compare it with 2 different sham controls: Bialosky et al. (2014) used a “standard” and an “enhanced” sham control. Three publications reported more than one trial: D'Souza et al. (2008) studied 2 groups with different types of headaches, and Assefi et al.'s (2008) publication included 2 active interventions and a matching sham control each. Finally, Sharpe et al. (2012) reported 2 trials in a single publication, which were treated entirely independently here. In general, only patients who informed the present analyses are counted in this table, patients were not counted twice, and analyses of reporting refer to individual trials.

* Intervention complexity: single-step or single-technique interventions were judged as “simple,” irrespective of how often these were applied, and others as complex. N = 194 unique trials.

### 3.2. Validation studies

Two^35,80^ of 8 validation studies^19,24,29,38,47,56^ were eligible for primary meta-analysis of pain-related outcomes. Three validation studies with more than 20 participants per group^24,29,56^ did not provide pain-related outcome data and were thus not included into respective meta-analyses. Howevere, they were included into the meta-analysis of trials reporting on blinding success.

### 3.3. Placebo and sham control intervention designs

Employed sham control interventions are listed and classified in the companion article, as are the results of the similarity assessment between sham and tested interventions and differences between therapies.^41^ Importantly, average similarity ratings were not significantly different between large and small trials (F(1,197) = 3.56, *P* = 0.061). However, physiotherapy/rehabilitation trials employed overall more dissimilar sham interventions than spinal manipulation trials, other manual therapies (excluding craniosacral therapy), and trials of spiritual or energetic therapies. Apart from the difference to other manual therapy trials, these differences were still significant when only large trials were tested and when pregnancy- and cancer-related trials were excluded as in our meta-regression analyses below.

### 3.4. Reports of blinding effectiveness

In 19 reports, blinding indices were provided or data were reported in a manner that allowed for calculating the Bang index.^4^ These studies were included for meta-analysis.

### 3.5. Reports of expectation of benefit from interventions

Only 6 trials (with 7 control interventions) reported expectancy data in a manner that allowed for data pooling.^10,11,28,29,56,60^ Apart from being an excessively small sample for meta-analysis,^13^ none of those studies reported significant differences in expectations between groups after an initial exposure to trial interventions. Meta-analysis and regression testing were thus not deemed promising and were not performed. In the remaining 23 trials with some mention of expectation-related assessments, the reasons for noncomparability were (1) inappropriate timepoint of assessments (either unexposed at baseline, or retrospectively after completing multisession treatment programme and thus likely confounded by satisfaction), (2) compound assessment with treatment credibility without individually reporting data of expectancy-only questions,^27^ (3) confounding expectancy and satisfaction, and (4) insufficient outcome data reporting. More details are presented in Supplementary Digital Content 4, available at http://links.lww.com/PAIN/B678.

### 3.6. Study outcomes

#### 3.6.1. Employed outcome measures

The most common unidimensional outcome measures employed were pain intensity rating scales (92%). Pressure pain thresholds were used in 8% of the 168 pairs of active and control interventions with extractable unidimensional outcome measures. Multidimensional outcome measures were available for 130 comparisons. These measures were mainly disability questionnaires (68%), followed by multidimensional pain questionnaires (19%), functional tests (8%), disease activity or symptom scores (5%), and general health questionnaires (2%). Relatively more objective and supposedly pain-related outcome measures were employed in 37% of all trials, including functional tests, disease markers, nerve conduction, autonomic nervous system parameters, brain imaging, work absenteeism, or medication use.

#### 3.6.2. Attrition

The difference in percentual attrition (ie, the differential attrition) between active and sham control groups was a mean of 0.4% more in active treatment groups (−0.74 to 1.5 95% CI, Q(136) = 6054, *P* = 0.51, *T*^2^ = 43.52, I^2^ = 97.8%), as estimated with a random-effects model with all studies weighted equally and applied to all large studies irrespective of therapy type (participants = 13,150, studies = 137, not reported in 6 studies). Longer studies reported more percentual attrition (*r*_s_'s(135) = 0.43, *P* < 0.001; 0.42, *P* < 0.001, respectively for active and control groups). Drop-out reasons related to the nature of the control intervention were reported in 9 studies (6.3%).

#### 3.6.3. Risk of bias

**Figure 2.**
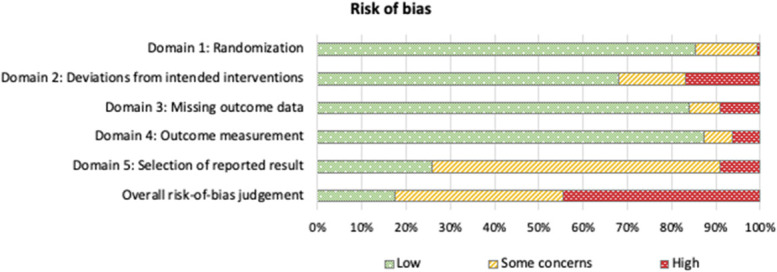
Risk of bias assessment for all comparisons with 20 or more participants per group (n = 128). Overall risk-of-bias was judged “High” if at least one domain had a rating of high risk-of-bias or if there were concerns in more than one domain. The overall rating of “Some concerns” was given if there were concerns in any one domain, and “Low” if all domains were rated as of low risk-of-bias, as per the Cochrane Risk of Bias tool 2 (RoB 2).^71^

In studies used for the primary meta-analysis, the overall risk of bias was judged low in 17% of studies, high in 44%, and some concerns existed for 38% (Fig. 2). In the smaller studies, 15% were rated as low risk and 82% high risk, and there were some concerns for 4% (not illustrated).

### 3.6.4. Pain-related outcome measures

Effect sizes (as standardised mean changes [SMC]) could be calculated for 166 intervention-vs-control comparisons for unidimensional outcomes and 125 comparisons using multidimensional outcome measures. Between-groups SMCs are illustrated below per subgroup. For the entire sample, between-group differences were not significantly different when studies were grouped by high, some or low overall risk of bias (unidimensional outcomes: F(2,164) = 1.217, *P* = 0.3.; multidimensional: F(2,123) = 0.231, *P* = 0.79).

### 3.7. Meta-analyses and meta-regression

Of 198 included comparisons, 120 were included in the primary meta-analysis, with a further 49 smaller studies used for secondary sensitivity analyses. Reasons for nonpooling included classification as outlier or apparent data errors (n = 5), patient populations in which no comparable improvements in pain report were expected (n = 8), and therapy subgroups too small for pooling (n = 16) (Fig. 1 – PRISMA flowchart). Because not all studies provided data for each outcome, study numbers in the following analyses vary.

### 3.7.1. Sham control within-group effects (placebo responses)

Across all studies included in the primary meta-analysis, changes from baseline to earliest follow-up within sham control groups had an average SMC effect size of −0.46 (−0.53 to −0.38 95% CI, *P* < 0.0001, *T*^2^ = 0.12, I^2^ = 69.9%, participants = 10.557, studies = 112) for unidimensional outcomes and −0.32 (−0.39 to −0.24 95% CI, *P* < 0.0001, *T*^2^ = 0.08, I^2^ = 62.06%, participants = 9.447, studies = 95) for multidimensional outcomes, indicating small to moderate placebo responses.

In the following, between-group differences will be presented per therapy subgroup and meta-regression analyses examining the role of different variables in predicting heterogeneity in trial outcomes.

### 3.7.2. Spinal manipulation subgroup

**Figure 3.**
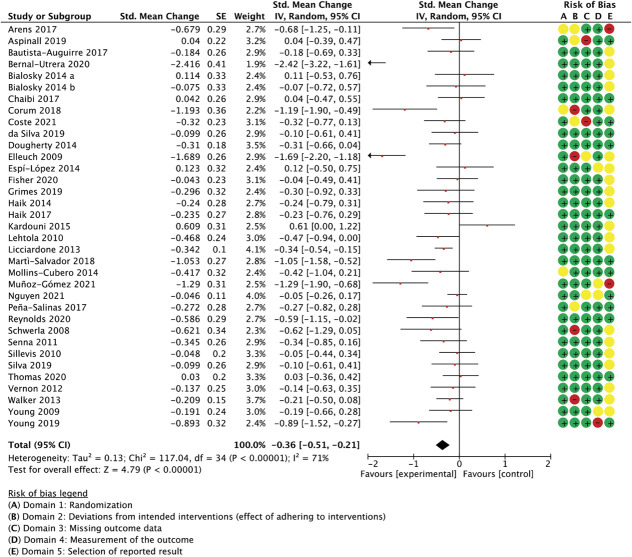
Spinal manipulation trials, unidimensional outcome measures as standardised mean changes with risk-of-bias assessment per study.

**Figure 4.**
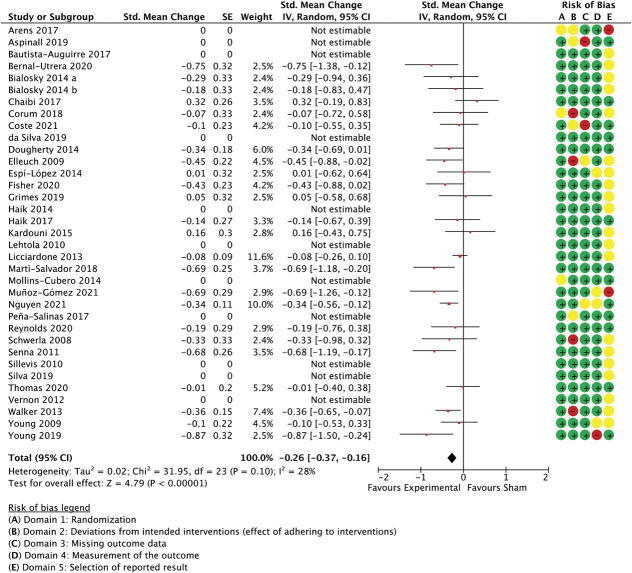
Spinal manipulation trials, multidimensional outcome measures as standardised mean changes with risk-of-bias assessment per study.

With 35 comparisons between an active treatment and a sham control, the overall effect in spinal manipulation trials was −0.36 (SMC) in favour of treatment groups for unidimensional pain measures (−0.51 to −0.21 95% CI, 3.084 participants, studies = 35, *T*^2^ = 0.14, I^2^ = 71.1%) (Fig. 3) and −0.26 for multidimensional measures (−0.37 to −0.15 95% CI, participants = 2.384, reported in 24 studies, *T*^2^ = 0.02, I^2^ = 26.7%) (Fig. 4).

A meta-regression model with the ratings for the following 3 features was able to predict 59% of the unaccounted between-study variance in unidimensional outcomes: similarity between active and control groups for the (1) number of treatment sessions, (2) the information about intervention efficacy provided to trial participants, and (3) treatment environment (Q[3,31] = 50.33, *P* < 0.0001, R^2^ = 0.59, participants = 3.084, studies = 35, residual *T*^2^ = 0.006, residual I^2^ = 10.1%) (Table 2). Differences in the number of sessions was the best predictor of differential outcomes. Exclusion of the 2 studies with the largest effect sizes^9,32^ rendered the model nonfunctional, making necessary the removal of the variable with the least variability (similarity in session number), which then resulted in a model with a reduced but significant predictive value (details not reported in table; Q(2,30) = 7.67, *P* = 0.02, R^2^ = 0.19, participants = 2.953, studies = 33, residual *T*^2^ = 0.006, residual I^2^ = 11.0%). A sensitivity analysis with all spinal manipulation therapy trials irrespective of sample size confirmed the validity of the complete model (details not reported in table; Q(3,38) = 43.86, *P* < 0.0001, R^2^ = 0.48, participants = 3.240, studies = 42, residual *T*^2^ = 0.02, residual I^2^ = 20.3%).

**Table 2:** Multiple meta-regression analysis, predicting between-study variance in unidimensional outcomes using the level of similarity between active and sham control groups for a number of selected features in spinal manipulation trials.

Descriptives							
Mean ES	Mean (SD) similarity rating (n of sessions)	Mean (SD) similarity rating (efficacy information)	Mean (SD) similarity rating (environment)	*R* ^2^	k		
−0.33	1.91 (0.37)	0.6 (1.03)	1.43 (0.74)	0.5934	35		

Homogeneity analysis							
	Q	df	*P*	*T* ^2^	I^2^		
Model	50.33	3	0.0000	0.077	94.0%		
Residual	34.49	31	0.30	0.006	10.1%		
Total	82.15	34	0.0000				

Regression coefficients							
	B	SE	−95% CI	+95% CI	Z	*P*	Beta
Constant	−2.64	0.36	−3.35	−1.93	−7.27	0.0000	0.0000
Similarity: N of sessions	1.09	0.19	. 72	1.47	5.75	0.0000	. 65
Similarity: efficacy information	0.13	0.05	0.02	0.23	2.3	0.02	0.27
Similarity: environment	0.07	0.08	−0.08	0.22	0.89	0.37	0.11
Method of moments random effects variance component							
v = 0.03							

Means and variance of the similarity ratings included in the model are provided, having a possible range of −2 (different for all studies) to 2 (similar across all studies).

This model was not able to predict a significant proportion of the variance when applied to between-group differences in multidimensional outcome measures, such as pain or disability questionnaires (Q(3,20) = 3.59, *P* = 0.31, *R*^2^ = 0.15, participants = 2.384, studies = 24, residual *T*^2^ = 0.00, residual I^2^ = 0%, results not shown in table), also not with small studies included as a sensitivity analysis (Q(3,24) = 3.6, *P* = 0.31, *R*^2^ = 0.13, participants = 2.488, studies = 28, residual *T*^2^ = 0.00, residual I^2^ = 0%). In this model, however, the (dis)similarity in treatment environment was clearly best able to predict outcomes. Simplification of the model to only include this variable improved its ability to account for between-study variance (Q(1,22) = 2.82, *P* = 0.09, *R*^2^ = 0.11, residual *T*^2^ = 0.00, I^2^ = 0%). It needs to be noted, however, that the unexplained variance in effect sizes was low for multidimensional outcomes (I^2^ = 28%, Fig. 4), leaving little scope for meta-regression analyses.

### 3.7.3. Craniosacral therapy trials

**Figure 5.**
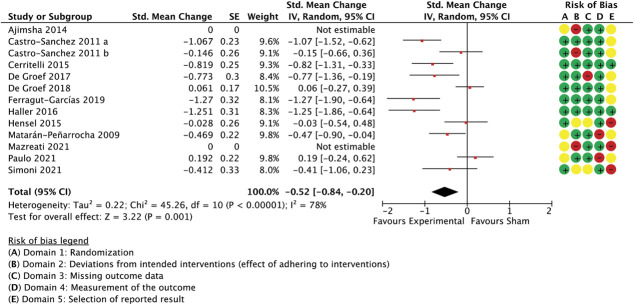
Trials of craniosacral therapies, unidimensional outcome measures as standardised mean changes with risk-of-bias assessment per study.

**Figure 6.**
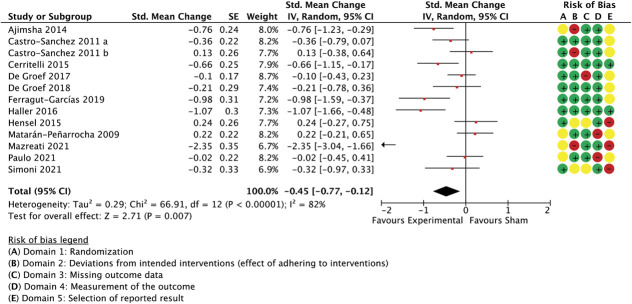
Trials of craniosacral therapies, multidimensional outcome measures as standardised mean changes with risk-of-bias assessment per study.

The meta-analysis included 13 studies of craniosacral or gentle myofascial interventions. Effect sizes compared with control interventions were −0.52 (−0.84 to −0.2 95% CI, *P* = 0.001, participants = 1.022, studies = 11, I^2^ = 78.1%) for unidimensional measures (Fig. 5) and −0.47 (−0.81 to −0.12 95% CI, *P* < 0.0001, participants = 1.162, studies = 13, I^2^ = 83.8%) for multidimensional ones (Fig. 6).

During meta-regression modelling for the differences in effects sizes in unidimensional outcomes between craniosacral trials, a model with the similarity ratings “body areas,” “application mode,” and “efficacy information” was found to predict 74% of the heterogeneity between studies (Table 3). Notably, directions of effects were such that trials showed smaller effects if control interventions consisted of devices (different application mode) and if participants were given different information about effectiveness of the study treatments. When similar body areas were treated in both groups, effect sizes were smaller too. In this subgroup, similarity in the number of sessions could not be used as predictor variable because all studies had the same number of treatment sessions between experimental and control groups. Adding 5 smaller studies reduced the usefulness of the model (details not reported in table; Q(3,12) = 3.35, *P* = 0.34, *R*^2^ = 0.15, residual *T*^2^ = 0.04, residual I^2^ = 37.9%, participants = 1.146, studies = 16).

**Table 3:** Multiple meta-regression analysis, predicting between-study variance in unidimensional outcomes using the level of similarity between active and sham control groups for a number of selected features in trials of craniosacral and gentle myofascial treatments.

Descriptives							
Mean ES	Mean (SD) similarity rating (body areas)	Mean (SD) similarity rating (efficacy information)	Mean (SD) similarity rating (application mode)	*R* ^2^	K		
−0.48	0.18 (1.9)	0.55 (0.93)	0.18 (2.1)	0.7299	11		

Homogeneity analysis							
	Q	Df	*P*	*T* ^2^	I^2^		
Model	19.25	3	0.0002	0.1	84.4%		
Residual	6.77	7	0.45	0.00	0%		
Total	26.02	10	0.0037				

Regression coefficients							
	B	SE	−95% CI	+95% CI	Z	*P*	Beta
Constant	−0.37	0.11	−0.6	−0.14	−3.18	0.0015	0.0
Similarity: body areas addressed	0.17	0.06	0.06	0.28	2.98	0.0028	0.62
Similarity: information about treatment effectiveness provided to participants	−0.33	0.14	−0.61	−0.05	−2.34	0.0195	−0.54
Similarity: application mode	−0.06	0.06	−0.18	0.06	−1.04	0.2984	−0.25
Method of moments random effects variance component							
v = 0.04							

Means and variance of the similarity ratings included in the model are provided, having a possible range of −2 (different for all studies) to 2 (similar across all studies).

For multidimensional outcome measures, the model predicted 38% of the between-studies variance (Q(3,9) = 7.0, *P* = 0.07, *R*^2^ = 0.38, residual *T*^2^ = 0.01, residual I^2^ = 21.6%, participants = 1.162, studies = 13, Table 4) and was confirmed in a sensitivity analysis with 2 additional smaller studies (details not reported in table; Q(3,11) = 7.54, *P* = 0.06, *R*^2^ = 0.37, residual *T*^2^ = 0.01, I^2^ = 15.2%, participants = 1209, studies = 15).

**Table 4:** Multiple meta-regression analysis, predicting between-study variance in multidimensional outcomes using the level of similarity between active and sham control groups for a number of selected features in trials of craniosacral and gentle myofascial treatments.

Descriptives							
Mean ES	Mean (SD) similarity rating (body areas)	Mean (SD) similarity rating (efficacy information)	Mean (SD) similarity rating (application mode)	*R* ^2^	k		
−0.46	0.38 (1.9)	0.46 (0.88)	0.46 (2.03)	0.3789	13		

Homogeneity analysis							
	Q	df	*P*	*T* ^2^	I^2^		
Model	7.0	3	0.07	0.021	57.1%		
Residual	11.48	9	0.24	0.013	21.6%		
Total	18.48	12	0.1				

Regression coefficients							
	B	SE	−95% CI	+95% CI	Z	*P*	Beta
Constant	−0.42	0.19	−0.79	−0.06	−2.25	0.02	0.0
Similarity: body areas addressed	0.08	0.1	−0.12	0.27	0.79	0.43	0.2
Similarity: information about treatment effectiveness provided to participants	0.07	0.22	−0.37	0.5	0.32	0.75	0.08
Similarity: application mode	−0.25	0.1	−0.44	−0.05	−2.47	0.02	−0.69
Method of moments random effects variance component							
v = 0.27							

Means and variance of the similarity ratings included in the model are provided, having a possible range of −2 (different for all studies) to 2 (similar across all studies).

### 3.7.4. Other manual therapy trials

**Figure 7.**
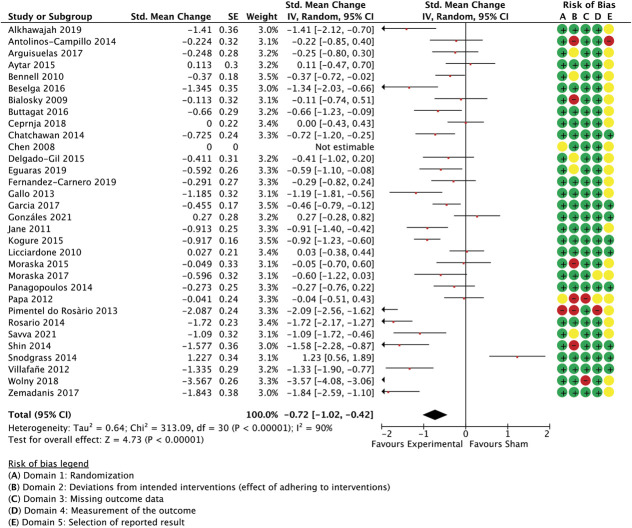
Other manual therapy trials (ie, excluding spinal manipulation and craniosacral techniques and including massage, articulation etc), unidimensional outcome measures as standardised mean changes with risk-of-bias assessment per study.

**Figure 8.**
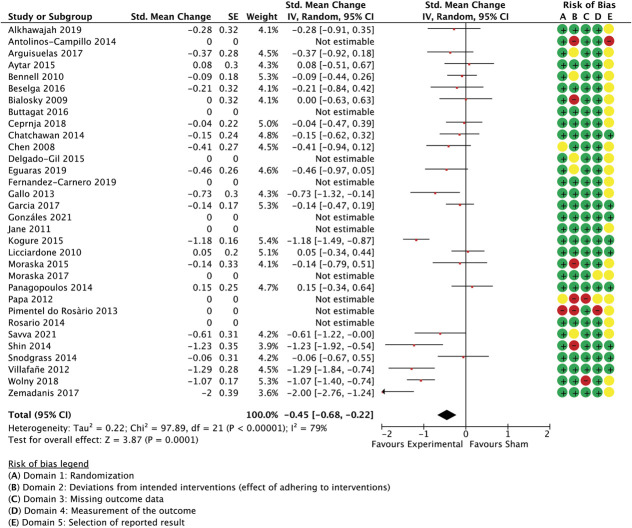
Other manual therapy trials, multidimensional outcome measures as standardised mean changes with risk-of-bias assessment per study.

Meta-analysed trials of other manual therapy encompassed 35 trials of massage, articulation, and manual therapies other than spinal manipulation and craniosacral or gentle myofascial techniques. The combined effect size was −0.72 (−1.02 to −0.42 95% CI, *P* < 0.0001, participants = 2.170, studies = 31, I^2^ = 90%) for unidimensional outcome measures (Fig. 7) and −0.45 (−0.68 to −0.22 95% CI, *P* < 0.0001, participants = 1.647, studies = 22, I^2^ = 79%) for multidimensional measures (Fig. 8).

When considering the regression model for this subgroup, it is worthwhile noting that certain similarity parameters had little variability, that is, ratings were consistently high across studies, and were thus unlikely to be of great predictive value in the model. These are “frequency of treatment” (1.97, 0.18 M, SD), “assessments” (1.97, 0.18), “delivery format” (1.97, 0.18), and “follow-up” (2.0, 0.0).

The model with the best fit included 3 covariates: similarity ratings between active and control for “number of sessions,” “efficacy information,” and “environment.” Nonetheless, this model only predicted 16.3% of the unexplained variance in between-group effect size differences measured in unidimensional outcomes (Table 5). To evaluate how dependent those findings were on the large between-group differences seen in some studies, all studies with confidence intervals that were not overlapping with those of the pooled effect^35,49,66,67,70,87^ were removed. This did not enhanced the model's predictive power (details not provided in table; Q(3,16) = 1.07, *P* = 0.78, *R*^2^ = 0.06, residual *T*^2^ = 0.01, residual I^2^ = 7.8%, participants = 1.587, studies = 25), highlighting the (dis)similarity in the treatment environment as the most important contributor to the model. A sensitivity analysis including the smaller studies provided similar results (Q(3,48) = 3.58, *P* = 0.31, *R*^2^ = 0.07, residual *T*^2^ = 0.00, residual I^2^ = 0%, participants = 2.752, studies = 52).

**Table 5:** Multiple meta-regression analysis, predicting between-study variance in unidimensional outcomes using the level of similarity between active and sham control groups for a number of selected features in other manual therapy trials.

Descriptives							
Mean ES	Mean (SD) similarity rating (number of sessions)	Mean (SD) similarity rating (efficacy information)	Mean (SD) similarity rating (environment)	*R* ^2^	k		
−0.72	1.81 (0.75)	0.58 (0.85)	1.61 (0.62)	0.1631	31		

Homogeneity analysis							
	Q	Df	*P*	*T* ^2^	I^2^		
Model	5.35	3	0.1482	0.00	43.9%		
Residual	27.43	27	0.4408	0.00	1.6%		
Total	32.78	30	0.3324				

Regression coefficients							
	B	SE	−95% CI	+95% CI	Z	*P*	Beta
Constant	−1.83	0.63	−3.07	−0.59	−2.9	0.0038	0.00
Similarity: environment	0.52	0.26	0.01	1.03	2.0	0.0457	0.36
Similarity: efficacy information	0.17	0.19	−0.19	0.54	0.92	0.3578	0.16
Similarity: number of sessions	0.1	0.22	−0.33	. 52	0.44	0.65	0.08
Method of moments random effects variance component							
v = 0.64							

Means and variance of the similarity ratings included in the model are provided, having a possible range of −2 (different for all studies) to 2 (similar across all studies).

For multidimensional outcome measures, the above model was better-able to predict between-study variance (Q(3,18) = 9.97, *P* = 0.019, *R*^2^ = 0.37, residual *T*^2^ = 0.0, residual I^2^ = 0%, participants = 1.647, studies = 22, Table 6), also in a sensitivity analysis with smaller studies that had multidimensional outcome measures available (Q(3,29) = 10.1, *P* = 0.017, *R*^2^ = 0.2, residual *T*^2^ = 0.03, residual I^2^ = 27.5%, participants = 1.957, studies = 33) or when an outlier was removed from the pool of large studies^88^ (Q (3,17) = 7.66, *P* = 0.05, *R*^2^ = 0.34, residual *T*^2^ = 0.00, I^2^ = 0%, participants = 1.607, studies = 21). In either case, the (dis)similarity in the number of sessions was the best predictor of between-study variance.

**Table 6:** Multiple meta-regression analysis, predicting between-study variance in multidimensional outcomes using the level of similarity between active and sham control groups for a number of selected features in other manual therapy trials.

Descriptives							
Mean ES	Mean (SD) similarity rating (number of sessions)	Mean (SD) similarity rating (efficacy information)	Mean (SD) similarity rating (environment)	*R* ^2^	k		
−0.37	1.7 (0.88)	0.5 (0.8)	1.6 (0.7)	0.37	22		

Homogeneity analysis							
	Q	Df	*P*	*T* ^2^	I^2^		
Model	9.97	3	0.0188	0.02	69.9%		
Residual	16.85	18	0.5338	0.00	0%		
Total	26.82	21	0.18				

Regression coefficients							
	B	SE	−95% CI	+95% CI	Z	*P*	Beta
Constant	−0.27	0.44	−1.13	−0.6	−0.61	0.54	0.0
Similarity: number of sessions	−0.34	0.14	−0.62	−0.05	−2.32	0.0203	−0.47
Similarity: environment	0.29	0.19	−0.08	0.66	1.56	0.12	0.31
Similarity: efficacy information	0.02	0.15	−0.27	0.32	0.17	0.87	0.03
Method of moments random effects variance component							
v = 0.22							

Means and variance of the similarity ratings included in the model are provided, having a possible range of −2 (different for all studies) to 2 (similar across all studies).

### 3.7.5. All manual therapy trials combined

Albeit a more heterogeneous group, we explored if the influential variables from the manual therapy subgroups also had predictive value when applied across spinal manipulation, craniosacral, and other manual therapy interventions. Across 77 studies with a total of 6.276 participants (SMC = −0.53, −0.68 to −0.39 95% CI, *P* < 0.0001, *T*^2^ = 0.22, I^2^ = 85.3%; combined forest plots not presented), a combined model with all 5 previously identified covariates predicted 16.9% of between-group variance in effect size, with only the similarity ratings for the number of treatment sessions and the treatment environment adding to the model's predictive power in a noteworthy fashion (Table 7). Including all smaller studies (participants total = 7.138, studies = 110), confirmed the model, again highlighting the outstanding impact on effect sizes when the number of treatment sessions or the treatment environment differ between active and sham control groups (Q(5,104) = 21.65, *P* = 0.0006, *R*^2^ = 0.15, residual *T*^2^ = 0.012, residual I^2^ = 13.9%). The predictive value of this model was similar for multidimensional outcome measures (Results not shown in figure or table. Large trials only, 59 studies, 5.193 participants; Meta-analysis of combined effect: SMC = −0.35, −0.47 to −0.23 95% CI, *P* < 0.0001, *T*^2^ = 0.15, I^2^ = 74.3; Meta-regression: Q(5,53) = 17.22, *P* = 0.004, *R*^2^ = 0.23, residual *T*^2^ = 0.005, residual I^2^ = 8.72%; Sensitivity analysis including small studies: Q(5,70) = 19.35, *P* = 0.0017, *R*^2^ = 0.18, residual *T*^2^ = 0.015, residual I^2^ = 20.4%, participants = 5.654, studies = 76).

**Table 7:** Multiple meta-regression analysis, predicting between-study variance in unidimensional outcomes using the level of similarity between active and sham control groups for a number of selected features in a combined sample of all large manual therapy trials.

Descriptives							
Mean ES	Mean (SD) similarity rating (number of sessions)	Mean (SD) similarity rating (environment)	Mean (SD) similarity rating (efficacy information)	Mean (SD) similarity rating (application mode)	Mean (SD) similarity rating (body areas addressed)	*R* ^2^	k
−0.53	1.88 (0.5)	1.47 (0.8)	1.1 (1.3)	0.34 (1.7)	1.4 (0.8)	0.169	77

Homogeneity analysis							
	Q	Df	*P*	*T* ^2^	I^2^		
Model	15.94	5	0.007	0.009	68.6%		
Residual	78.24	71	0.26	0.006	9.3%		
Total	94.18	76	0.077				

Regression coefficients							
	B	SE	−95% CI	+95% CI	Z	*P*	Beta
Constant	−1.57	0.31	−2.18	−0.95	−5.0	0.0000	0.0
Similarity: number of sessions	0.33	0.15	0.04	0.62	2.3	0.0239	0.24
Similarity: environment	0.28	0.1	0.09	0.47	2.92	0.0035	0.31
Similarity: efficacy information	0.09	0.08	−0.07	0.25	1.13	0.26	0.12
Similarity: application mode	−0.04	0.04	−0.12	0.05	−0.85	0.39	−0.09
Similarity: body areas	−0.03	0.05	−0.14	0.07	−0.63	0.53	−0.31
Method of moments random effects variance component							
v = 0.33							

Means and variance of the similarity ratings included in the model are provided, having a possible range of −2 (different for all studies) to 2 (similar across all studies).

### 3.7.6. Physical therapy interventions, rehabilitation, and exercise

**Figure 9.**
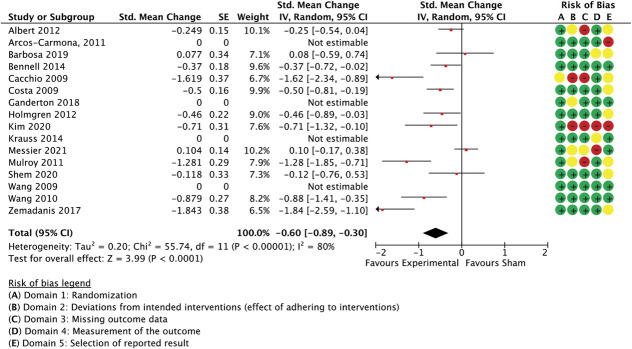
Physical therapy and rehabilitation intervention trials. Between-group differences in unidimensional outcome measures are presented as standardized mean change scores alongside risk-of-bias assessments per study.

**Figure 10.**
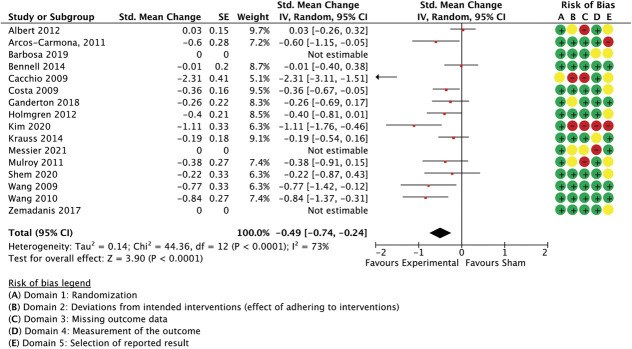
Physical therapy and rehabilitation intervention trials. Between-group differences in multidimensional outcome measures are presented as standardized mean change scores alongside risk-of-bias assessments per study.

Sixteen studies with a total of 1.554 participants examined rehabilitation and exercise interventions. Aggregated effect sizes were −0.6 (−0.89 to −0.3 95% CI, *P* < 0.0001, I^2^ = 80%) for unidimensional measures (Fig. 9) and −0.49 (−0.74 to −0.24 95% CI, *P* < 0.0001, I^2^ = 73%) for multidimensional outcomes (Fig. 10).

Surprisingly, in this subgroup, similarity regarding the extent of treatment exposure (“number of sessions”) was not found to reliably inform a potential regression model to predict between-group effect sizes, although large variation existed between studies in how similar active and control groups were for the amount of treatment received; differences in the treatment environment were also not predictive. Instead, (dis)similarity in treatment individualisation and the level of fidelity monitoring predicted 86% of between-group variance in unidimensional outcomes (Table 8). This was confirmed when adding 3 studies with less than 20 participants per arm (Q(2,11) = 13.9, *P* = 0.001, *R*^2^ = 0.61, residual *T*^2^ = 0.0, residual I^2^ = 0.0%, participants = 1.251, studies = 15). The small number of studies in this subgroup prevented the addition of further variables to a single model.

**Table 8:** Multiple meta-regression analysis, predicting between-study variance in unidimensional outcomes using the level of similarity between active and sham control groups for a number of selected features in a sample of all large physiotherapy and rehabilitation exercise trials.

Descriptives							
Mean ES	Mean (SD) similarity rating (individualisation)	Mean (SD) similarity rating (fidelity monitoring)	*R* ^2^	k			
−0.46	−0.17 (1.3)	1.0 (1.1)	0.62	12			

Homogeneity analysis							
	Q	Df	*P*	*T* ^2^	I^2^		
Model	12.28	2	0.0022	0.048	83.7%		
Residual	7.67	9	0.5678	0.00	0%		
Total	19.95	11	0.046				

Regression coefficients							
	B	SE	−95% CI	+95% CI	Z	*P*	Beta
Constant	−0.73	0.17	−1.07	−0.39	−4.23	. 0000	0.0
Similarity: individualisation	−0.24	0.11	−0.45	−0.3	−2.2	0.0276	−0.56
Similarity: fidelity monitoring	0.18	0.13	−0.07	0.42	1.39	0.1647	0.35
Method of moments random effects variance component							
v = 0.11							

Means and variance of the similarity ratings included in the model are provided, having a possible range of −2 (different for all studies) to 2 (similar across all studies).

There were more studies that provided multidimensional outcome measures than unidimensional outcomes in the rehabilitation and exercise group, resulting in 14 large trials for this analysis (1.257 participants). The same model with similarity in fidelity monitoring and treatment individualisation predicted a nonsignificant proportion (7%) of the variance in between-group differences (Q(2,11) = 1.1, *P* = 0.57, *R*^2^ = 0.1, residual *T*^*2*^ = 0.015, residual I^2^ = 25.1%, no table provided). The model was further weakened by removing an outlier^20^ (Q(2,10) = 0.0, *P* = 1.0, *R*^2^ = 0.00, residual *T*^2^ = 0.00, residual I^2^ = 2.7%). Adding the 2 small trials available in this group made little difference (Q(2,12) = 1.14, *P* = 0.57, *R*^2^ = 0.08, residual *T*^2^ = 0.01, I^2^ = 20.8%, participants = 1.314, studies = 16). Similarity in the number of treatment sessions or treatment environment were again not found to provide any predictive value.

### 3.7.7 Psychological interventions

**Figure 11.**
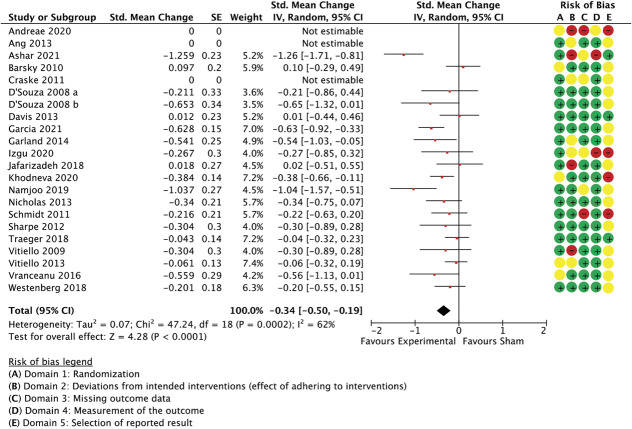
Psychological and behavioural interventions. Between-group differences in unidimensional outcome measures are presented as standardized mean change scores alongside risk-of-bias assessments per study.

**Figure 12.**
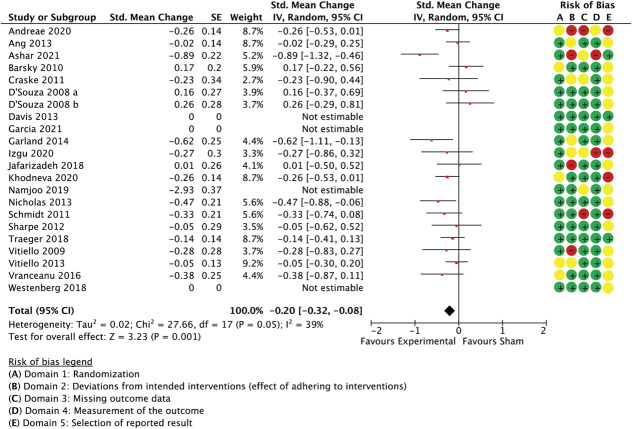
Psychological and behavioural interventions. Between-group differences in multidimensional outcome measures are presented as standardized mean change scores alongside risk-of-bias assessments per study. The study by Namjoo et al.^59^ is illustrated here but was excluded from the meta-analysis as an outlier (weight in the model = 0%).

Nineteen meta-analysed large trials studied psychological or behavioural interventions and employed unidimensional outcomes (effect size −0.34 [−0.50 to −0.19 95% CI], *P* < 0.0001, I^2^ = 62%, participants = 2.085, Figure 11) and multidimensional measures (−0.2 [−0.32 to −0.08 95% CI], *P* = 0.001, I^2^ = 39%, participants = 2.122, studies = 18, Figure 12). One study^59^ was removed as an outlier before meta-analysis of multidimensional outcomes.

A multiple meta-regression model with the variables “application mode” and “attention focus/cognitive function” predicted 41.3% of the remaining variance between studies in unidimensional outcomes (Table 9). The model also predicted between-study variance when a small study was added (Q(2,17) = 11.69, *P* = 0.0029, *R*^2^ = 0.41, residual *T*^2^ = 0.00, I^2^ = 0.3%, participants = 2.119, studies = 20).

**Table 9:** Multiple meta-regression analysis, predicting between-study variance in unidimensional outcomes using the level of similarity between active and sham control groups for a number of selected features in large trials of psychological and behavioural interventions.

Descriptives							
Mean ES	Mean (SD) similarity rating (application mode)	Mean (SD) similarity rating (attention focus/cognitive function)	*R* ^2^	k			
−0.33	0.84 (1.7)	−0.26 (1.6)	0.41	19			

Homogeneity analysis							
	Q	Df	*P*	*T* ^2^	I^2^		
Model	11.48	2	0.0032	0.02	82.6%		
Residual	16.3	16	0.4222	0.00	1.8%		
Total	27.78	18	0.0654				

Regression coefficients							
	B	SE	−95% CI	+95% CI	Z	*P*	Beta
Constant	−0.4	0.08	−0.55	−0.24	−4.97	0.0000	0.0
Similarity: attention	0.09	0.04	0.006	0.17	2.1	0.036	0.41
Similarity: application mode	0.09	0.04	0.002	0.17	2.	0.045	0.41
Method of moments random effects variance component							
v = 0.03							

Means and variance of the similarity ratings included in the model are provided, having a possible range of −2 (different for all studies) to 2 (similar across all studies).

The same model predicted 54% of unexplained heterogeneity in multidimensional outcome measures (Table 10), confirmed by a sensitivity analysis with an additional small study (Q(2,17) = 6.92, *P* = 0.031, residual *T*^2^ = 0.03, residual I^2^ = 39.9%, participants = 2.241, studies = 20).

**Table 10:** Multiple meta-regression analysis, predicting between-study variance in multidimensional outcomes using the level of similarity between active and sham control groups for a number of selected features in large trials of psychological and behavioural interventions.

Descriptives							
Mean ES	Mean (SD) similarity rating (application mode)	Mean (SD) similarity rating (attention focus/cognitive function)	*R* ^2^	k			
−0.3	0.95 (1.5)	−0.26 (1.6)	0.21	19			

Homogeneity analysis							
	Q	Df	*P*	*T* ^2^	I^2^		
Model	7.18	2	0.0276	0.01	72.1%		
Residual	27.53	16	0.036	0.03	41.9%		
Total	34.71	18	0.0103				

Regression coefficients							
	B	SE	−95% CI	+95% CI	Z	*P*	Beta
Constant	−0.49	0.13	−0.73	−0.24	−3.84	0.0001	0.0
Similarity: attention	0.02	0.06	−0.1	0.15	0.36	0.7219	0.06
Similarity: application mode	0.18	0.07	0.04	0.31	2.515	0.0109	0.44
Method of moments random effects variance component							
v = 0.13							

Means and variance of the similarity ratings included in the model are provided, having a possible range of −2 (different for all studies) to 2 (similar across all studies).

### 3.7.8. Blinding index subgroup

The Bang blinding index could be calculated for 18 comparisons between an active and a control intervention. In this subgroup, there were 9 spinal manipulation, 7 other manual therapy trials and one trial each of physiotherapy/rehabilitation and spiritual healing (Reiki). The average sample size at randomization was 64.4 patients (SD 37.4, range 10-154).

**Figure 13.**
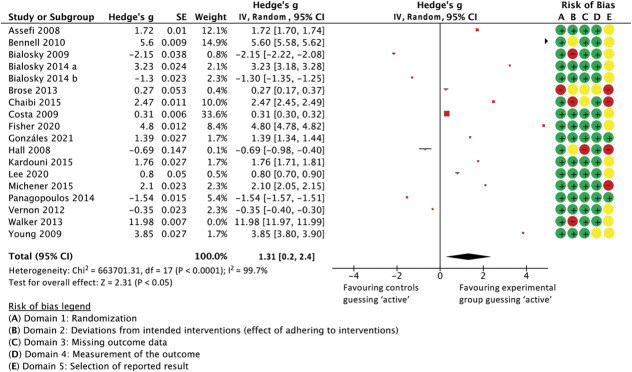
Forest plot showing all studies for which the Bang BI could be calculated. The ratio between BI in active and control groups is presented as Hedge's *g*, with values near 0 indicating that participants in both groups were likely to make similar guesses as to which treatment they received (ie, were adequately blinded). Values below 0 indicate that patients in the sham arm believed disproportionately more that they were in the active arm than those in the actual active group (indicating higher sham credibility), and values to the right indicating that the active treatment made more patients think that they had a real treatment than the sham treatment in the same study (in other words, fewer sham-arm patients believed that theirs was a real treatment than active-arm patients did about their intervention, indicating unsuccessful blinding). An extreme outlier (Walker et al., 2013)^69^ is shown in the forest plot but does not feed into the meta-analysis (weight = 0%).

As per Colagiuri et al.,^25^ the blinding indices of each trial's 2 groups were combined as a ratio using Hedge g, with values larger than 0 indicating that participants in the active group were more likely to correctly guess their allocation to the active group than those in the control group, and values below 0 indicating that patients in the sham control group were more likely to wrongly guess that they received an active treatment compared to those in the active group. Having excluded one outlier where blinding was fully unsuccessful,^81^ the combined Hedge g was 1.31 favouring participants in the experimental groups guessing correctly over control participants believing to have received active treatment (unsuccessful or unbalanced blinding [0.2 to 2.43 95% CI, *P* = 0.02, I^2^ = 99.7%, participants = 1.013, studies = 17]). Data are presented as a forest plot below (Fig. 13).

A meta-regression model with the similarity variables “sensation” and “application mode” (eg, manual vs device) was able to predict 35% of the unexplained variance between studies (Q(2,14) = 8.92, *P* = 0.012, *R*^2^ = 0.35, residual *T*^2^ = 0.003, residual I^2^ = 15.1%, participants = 1.013, studies = 17, Table 10). Notably, this model was primarily driven by differences in the sensations participants were exposed to. This effect would have been reverted to an emphasis on differences in application modes by including the Walker et al.^81^ study (participants = 183), where detuned ultrasound, a hand-held device delivering low-force impulses to paraspinal tissues, and random hand placements on the patient's back were used as a sham control for individualised chiropractic treatments (Q(2,15) = 12.0, *R*^2^ = 0.54, *P* = 0.002, residual *T*^2^ = 0.00, residual I^2^ = 0%). The apparent importance of choosing similar application modes and producing similar sensations in control interventions to achieve balanced blinding was further emphasised by excluding another study with largely unsuccessful blinding: Bennell et al. (2010)^8^ used detuned ultrasound and a nontherapeutic gel to control for standardised manual therapy and a home exercise programme for shoulder pain in 120 participants (Q(2,13) = 2.25, *R*^2^ = 0.14, *P* = 0.32, residual *T*^2^ = 0.001, residual I^2^ = 4.8%, participants = 893, studies = 16). It is also noteworthy that the trials in this subgroup exposed participants in either group to the same extent of treatment (eg, “number of sessions”) and treatment environment, so that these variables could not be tested for Table 11.

**Table 11:** Multiple meta-regression analysis, predicting between-study variance in the success of blinding using the level of similarity between active and sham control groups for a number of selected features in all trials where Bang blinding index could be calculated (8 spinal manipulation trials, 7 other manual therapy, 1 physiotherapy and rehabilitation exercise, and 1 Reiki intervention).

Descriptives							
Mean ES	Mean (SD) similarity rating (sensations)	Mean (SD) similarity rating (application mode)	*R* ^2^	k			
1.32	1.24 (1.15)	1.71 (0.99)	0.35	17			

Homogeneity analysis							
	Q	df	*P*	*T* ^2^	I^2^		
Model	8.92	2	0.0116	0.008	77.6%		
Residual	16.48	14	0.2847	0.003	15.1%		
Total	25.4	16	0.0631				

Regression coefficients							
	B	SE	−95% CI	+95% CI	Z	*P*	Beta
Constant	3.0	0.87	1.27	4.7	3.42	0.0006	0.0
Similarity: sensations	−0.97	0.52	−2.0	0.05	−1.87	0.06	−0.5
Similarity: application mode	−0.28	0.6	−1.46	0.91	−0.46	0.65	−0.12
Method of moments random effects variance component							
v = 3.09							

Means and variance of the similarity ratings included in the model are provided, having a possible range of −2 (different for all studies) to 2 (similar across all studies).

In 15 trials (1.084 participants), both the Bang BI and pain-related outcome data were available (estimated aggregated effect size for unidimensional outcomes: −0.22 [−0.39 to −0.04 95% CI, *P* = 0.015, *T*^2^ = 0.05, *I*^2^ = 43.7%]; multidimensional outcomes: −0.23 [−0.36 to −0.09 95% CI, *P* = 0.001, *T*^2^ = 0.00, *I*^2^ = 9.1%], participants = 1.019, studies = 14). Meta-regression with the BI ratio as a potential predictor of between-group differences showed no relationship between the 2 for unidimensional outcome measures (Q(1,13) = 0.01, *R*^2^ = 0.001, *P* = 0.9) or multidimensional outcomes (Q(1,12) = 1.5, *R*^2^ = 0.12, *P* = 0.23), which was unsurprising given the small between-study heterogeneity in outcomes.

### 3.7.9. Differential attrition and trial outcomes

Differential attrition did not predict significant between-study variance in trial outcomes in the combined sample for unidimensional outcomes (Q(1,148) = 3.2, *R*^2^ = 0.02, *P* = 0.07, residual *T*^2^ = 0.02, residual I^2^ = 26.4%, participants = 11.009, studies = 150) or multidimensional measures (Q(1,115) = 0.0, *R*^2^ = 0.0, *P* = 0.9, residual *T*^2^ = 0.03, residual I^2^ = 31.3%, participants = 9.868, studies = 117).

### 3.7.10. Differential attrition and similarity between active and sham control interventions

In a random-effects meta-regression model with all studies weighted equally, a model with 6 similarity ratings was able to significantly predict 12.7% of the variation in differential drop-outs, with differences in fidelity monitoring contributing most to the model's predictive value (Table 12). A sensitivity analysis with all studies irrespective of sample size confirmed the model, with differences in treatment environment now increasing their contribution to the model (Q(6,143) = 13.2, *R*^2^ = 0.08, *P* = 0.04, residual *T*^2^ = 0.0, residual I^2^ = 0.0%, participants = 11.829, studies = 150, no detail provided in table).

**Table 12:** Multiple meta-regression analysis, predicting between-study variance in the level of differential attrition using the level of similarity between active and sham control groups for a number of selected features.

Descriptives							
Mean (SD) similarity ratings	n of sessions	Standardisation	Fidelity monitoring	Individualisation	Application mode	Participation	Treatment environment
	1.69 (0.9)	0.97 (1.2)	0.61 (1.0)	0.5 (1.5)	0.8 (1.8)	0.9 (1.5)	1.2 (1.1)
Mean ES				*R* ^2^	k		
0.3068				0.127	111		

Homogeneity analysis							
	Q	df	*P*	*T* ^2^	I^2^		
Model	15.1	6	0.0197	0.08	60.2%		
Residual	104.	104	0.48	0.00	0%		
Total	119.1	110	0.26				

Regression coefficients							
	B	SE	−95% CI	+95% CI	Z	*P*	Beta
Constant	−1.13	1.5	−4.1	1.83	−0.75	0.45	0.0
Similarity: fidelity monitoring	−2.14	0.68	−3.5	−0.8	−3.13	0.0017	−0.3
Similarity: application mode	0.44	0.45	−0.45	1.32	1.0	0.34	0.12
Similarity: environment	0.87	0.7	−0.51	2.25	1.23	0.22	0.13
Similarity: N of sessions	0.62	0.86	−1.06	2.29	0.72	0.47	0.08
Similarity: standardisation	0.57	0.59	−0.59	1.72	0.96	0.34	0.1
Similarity: participation	−0.16	0.53	−1.19	0.88	−0.29	0.77	−0.03
Method of moments random effects variance component							
v = 46.5							

Means and variance of the similarity ratings included in the model are provided, having a possible range of −2 (different for all studies) to 2 (similar across all studies).

## 4. Discussion

Analysing 194 publications, we found moderate placebo responses across physical, psychological, and self-management therapies for pain, with patients in sham control groups improving with an average effect size of 0.46 from pretreatment to the first posttreatment assessment. Benefits of experimental interventions over sham controls ranged from effect sizes of 0.34 in psychological interventions (number needed to treat, NNT ≈ 9) to 0.72 in some manual therapies (NNT ≈ 4),^53^ with risk-of-bias concerns in most trials. Effect sizes were smaller when multidimensional measures such as disability questionnaires were employed, as opposed to simple pain intensity scales.

In most of the studied intervention subgroups, there was considerable unexplained heterogeneity in trial results between studies. Assessing and rating the level of similarity between experimental and sham control interventions, however, explained some of that heterogeneity. For example, a proportion of variance in trial outcomes was explained by how different tested and control interventions were regarding the number of treatment sessions, application modes, or treatment environment. Furthermore, trials were at a higher risk of differential attrition when there were differences in monitoring of the groups' treatment adherence. In a subgroup of studies where the blinding success of control interventions had been measured, meta-regression analyses were also able to partially predict the risk of unbalanced blinding.

These findings underline the importance of carefully matched experimental and control interventions in efficacy and mechanistic trials. They further provide quantitative evidence that resemblance in some features may be particularly influential. We identified several features that are commonly assessable from trial reports, capture influential aspects of (dis)similarity, and have little conceptual or practical overlap between each other. These features are the number of treatment sessions, application mode, intervention individualisation, fidelity monitoring, and the treatment environment. These are presented and discussed in Table 13.

**Table 13:** Overview of sham control design features that were shown to have an impact on trial outcomes.

Control intervention feature and description	Influence on trial results and discussion
Number of treatment sessions *The number of times a patient receives the interventions, both through a provider or self-delivered.*	In our preliminary assessments, we found this feature to be highly correlated with, eg, treatment duration and treatment frequency. We thus suspect the number of treatment sessions to be a good proxy for the *extent of treatment* received by patients. It is important to note that differences in the exposure to an intervention often occur not only when patients are asked to attend more clinic appointments than those in the control group but also when they are provided with home exercise or self-management programmes that expose them to higher “dosages” of specific and contextual effects.
Application mode *The mode or medium through which an intervention is delivered, including telephone, online, hands-on or conversation-based delivery. In this sample, device-based active treatments were not eligible for review, but device-based shams (such as detuned ultrasound) were common.*	Questioning the suitability of sham devices for blinding in non-device RCTs, differences in the “tool” through which active and control interventions are applied lead to less credible control interventions. Regarding effects on trial outcomes, the effect can likely go either way, with more elaborate sham controls leading to smaller between-group differences^33^ or undermining through supposed credibility differences. In other words, sham devices do seem to produce comparable placebo effects to respective active interventions but may compromise trials in other ways. The use of sham devices is still common, especially in physiotherapy and nonspinal manipulation trials (see Ref. 41). In psychological and behavioural interventions, the “application mode” may differ when control interventions rely on print or web-based materials rather than personal interactions.
Intervention individualisation *The extent to which treatments are personalised or adapted to each patient may reach from fully standardised to highly individualised.*	Likely somewhat overlapping with the concept of treatment standardisation, we found that the extent to which the active and control interventions are individualised to patients can influence trial outcomes. We can only suspect that the individualisation is communicated to the patient verbally and nonverbally, making the patient feel valued more or suppose that the treatment addresses the root cause of their problem. Contrastingly, patients receiving generic control treatment may wonder why symptomatic areas were not touched or topics not addressed that felt relevant to them. Highly standardised interventions of course offer little room for individualisation.
Patient participation *The level of patients contributing to the intervention, ranging from physically and psychologically passive recipients to largely shaping the intervention themselves.*	Many commonly used control interventions are more passive than the study treatment, especially in physiotherapy, self-management, and exercise trials. Our regression models did not clearly indicate, however, whether different participation levels contribute to differences in pain reports. While patients play an active role in most cognitive, behavioural, and exercise-based interventions for pain, manual therapies can be passive experiences for patients. This allows researchers to employ passive control interventions that match a passive treatment, as reflected by high similarity ratings in this subgroup.
Fidelity monitoring *Monitoring and potentially promoting treatment adherence by patients or therapists' delivery of interventions according to protocols.*	When the treatment adherence or therapists' intervention provision are ensured more in one group than in the other, patients will likely receive different amounts of active and control care. Differences in fidelity monitoring between groups was influential for differential attrition across all trials. Not only in physiotherapy but also in behavioural interventions, patients often perform (parts of) the intervention under their own supervision, eg, as home-based exercise programmes. Therefore, fidelity monitoring is more relevant than, eg, in most manual therapy scenarios. Potentially, fidelity monitoring itself also acts as an additional contextual factor, ensuring that patients believe that it is important how and how often the intervention is performed.
Treatment environment *The physical environment in which interventions take place.*	One of the most consistent predictors of between-study variance in pain outcomes, the treatment environment differs, eg, when the active intervention is mainly performed in a healthcare centre and the control intervention at the patient's home or vice versa.^58^ Another example is the study by Arcos-Carmona et al. (2011) where the intervention involved aerobic exercise in a swimming pool while the control group received a magnetotherapy sham, lying prone on a treatment bench.^2^ Unsurprisingly, this difference in environment comes with large differences in contextual factors that may result in differential placebo effects. While treatment environments were usually similar for all studied therapy types, the consistency with which differences in this feature predicted trial outcomes shows that matching of treatment environments ought to be paramount.

The meta-analysis of similarity features was complicated by insufficient reporting, possibly explaining why some features that are commonly deemed important to be matched between study groups did not appear significant in our analyses. This may apply to the (dis)similarity in cointerventions and concomitant treatments^36^ and personal interactions with staff and providers.^5,43^ The information provided to participants about the supposed efficacy or rationale of the interventions is also rarely reported or must be inferred. Commonly identified as important in the literature,^17,18,55,77^ our analyses were nonetheless sensitive to this feature. Furthermore, we were unable to ascertain whether the use of different providers for both groups changed trial results because most trials used the same providers or did not report this information.^41^ In addition, there are therapy-specific considerations that cannot be reliably captured in a systematic evidence synthesis, for example, the replication of treatment side effects in the control group or the modification of providers' treatment “styles” to individual patients.

Inconsistency in our meta-regression findings may further be linked to the lack of variability in similarity ratings within a given subgroup, little unexplained heterogeneity in pain-related outcomes, or, of course, the possibility that similarity for a given item did not influence effect sizes sufficiently to detect a link. Relatedly, the patients' experience may be dominated by different treatment aspects depending on therapy type, possibly explaining why certain features only significantly predicted study results in individual therapy types. Finally, it could be argued that this review's results may be influenced by publication bias, with negative results less likely to be published.^65^ However, missing small trials will not have impacted our primary analyses (as these only included trials with more than 20 participants per arm). While we are unable to estimate the impact of missing large trials, we have not made judgements on treatment efficacy so that testing for publication bias was not deemed necessary.

Some of the employed similarity ratings may overlap with supposed “specific” elements of treatments. If, for example, cognitive distraction is a purportedly integral part of the intervention, then of course the similarity rating will be low for this feature and links to trial outcomes may be found. This may have influenced our findings in the subgroup of psychological and behavioural interventions. In this instance, the question of similarity becomes a mechanistic one, demonstrating that treatment mechanisms need to be considered in the design of control interventions and ideally reported. Conversely, mechanisms can potentially be studied using our meta-analytic approach of assessing the predictive role of (dis)similarity between active and control interventions for specific features.

Further important insights of our review relate to the nature of pain-related outcomes employed in RCTs. First, distinguishing unidimensional from multidimensional outcomes enabled us to demonstrate that the latter, more complex outcome measures produce less between-study variance in results, leading to more consistent but smaller effects. Similarly, placebo responses were smaller for multidimensional outcomes. Second, multidimensional outcomes appeared less susceptible to contextual effects produced by differences between active and control interventions. In other words, they may allow for less well-matched control interventions. However, this statement needs to be cautioned because we do not know if there are unidentified confounding factors and because this effect is partly due to the reduced heterogeneity just discussed, weakening our meta-regression models for multidimensional outcome measures.

Apart from the similarity between active and control interventions in efficacy trials, we should consider other sources of heterogeneity in trial outcomes. Patients expectations of benefit regarding the study treatment and the planned number of provider interactions have been identified as predictors of the placebo response in drug trials.^69,79^ Both variables are conceptually related to some of the predictors identified in the present review. Other, psychobiological predictors are known but have not been tested here, including baseline pain, the nature of the studied painful condition, gender, patient personality traits, and different healthcare settings.^40,51,55,79^ Of course, differences in intervention efficacy will have contributed to heterogeneity in effect sizes in our, often diverse, intervention subgroups. The same applies to trial-specific risk of bias, which we illustrated but did not formally include in our modelling. To these known predictors of trial outcomes, we have added the insight that resemblance between test and control interventions matters.

It needs to be noted that the prominent role of blinding in clinical trial research has recently been questioned in opinion articles^1^ and by a meta-analysis that did not find differences in outcomes between blinded and nonblinded studies.^57^ Our own subgroup analysis in studies reporting on blinding effectiveness indicated a similar direction but small numbers and little between-study heterogeneity prevent firm conclusions. Conversely, our other analyses clearly demonstrated that trial outcomes partly depended on characteristics of control interventions and their similarity to experimental treatments. While this testing did not consider patients' blinding status, a possible mediator of this finding was the placebo effect, rather than knowledge of group allocation. This is supported also by the fact that features known to drive placebo responses were most predictive, namely, the extent of therapeutic interaction, treatment environment, and other features directly affecting the patient experience. Differences in application modes were predictive of pain-related results in some cases, possibly because of differences in placebo responses or because they facilitated unblinding, as was the case in a subgroup of trials where blinding effectiveness was reported. To gain more certainty about the influence of blinding success on trial outcomes, more consistent reporting of blinding effectiveness is required. Nonetheless, we demonstrated that successful blinding is more likely to be achieved with control interventions that resemble experimental treatments. Participant blinding in trials is likely influenced by factors rarely assessable from trial reports, such as staff's compliance with trial procedures, or contact among participants recruited from the same population. Participant blinding is even more challenging in PPS pragmatic and comparative effectiveness trials and is rarely conducted: less than one-quarter of these perform participant blinding.^42^

Considering our findings and what is known about the power of placebo effects in the absence of blinding (“open-label placebos”),^12,22,50,82^ it appears that well-matched control interventions are mainly important to prevent skewed trial results in explanatory trials based on different levels of psychosocial contextual factors. Whether this is independent of blinding effectiveness requires further investigation. A likely mediator of the placebo effect in trials are participants' expectations of benefit.^63^ Here also, our unsuccessful attempt to compare reports of expectancies highlights a need for homogenisation of methods and reporting. The concept needs to be clearly delineated from treatment credibility and satisfaction, appropriate timepoints and methods of expectancy assessment in trials agreed, and outcome data reported.^27^

## 5. Conclusions

The present review provides quantitative support for the recommendation that experimental and control interventions in efficacy and mechanistic trials should be “structurally equivalent”^52,64^ or “indistinguishable”.^5^ Crucially, this review added the insight that similarity in the extent of intervention exposure, treatment environment, and patient experience are the most important considerations. Across different groups of physical, psychological, and self-management interventions, these factors predicted variability in trial results. Differences in these and several other, sometimes therapy-specific considerations can lead to differences in contextual effects and thus biased trial outcomes. Apart from impacting pain-related outcomes, such differences can undermine participant blinding and promote differential attrition. More work is needed to quantify the effects of blinding failure on pain outcomes, suggesting that there is an urgent need to conduct routine assessments of blinding effectiveness in clinical trials. Efforts to promote adequate reporting of control interventions, such as the TIDieR-Placebo checklist,^44^ are welcomed. Nonetheless, more work is required to translate the present findings into evidence-based recommendations for the design, testing, and conduct of control interventions in efficacy and mechanistic RCTs of complex physical, psychological, and self-management interventions for people with pain.

## Conflict of interest statement

Mr Hohenschurz-Schmidt reports support through a PhD Studentship from the Alan and Sheila Diamond Trust for this work and personal fees from Altern Health Ltd, outside the submitted work; Dr. Draper-Rodi reports grants from Alan and Sheila Diamond Charitable Trust, during the conduct of the study; Dr. Scott reports grants from Medical Research Council and Versus Arthritis, and from the National Institute for Health and Care Research, outside the submitted work; Dr. Vollert reports personal fees from Vertex Pharmaceuticals and personal fees from Embody Orthopaedic, outside the submitted work; Prof Rice reports personal fees from IMMPACT and grants from the Alan and Sheila Diamond Trust during the conduct of the study, and personal fees from Imperial Consultants, personal fees from MD Anderson Cancer Center, other from spinifex, other from Medicines and Healthcare products Regulatory Agency (MHRA), and Commission on Human Medicines ‐ Neurology, Pain & Psychiatry Expert Advisory Group, all outside the submitted work; In addition, Dr. Rice has a patent WO 2005/079771 & a patent EP13702262.0/ WO2013 110945 pending. All other authors report that they have no conflicts of interest.

## Appendix A. Supplemental digital content

Supplemental digital content associated with this article can be found online at http://links.lww.com/PAIN/B675, http://links.lww.com/PAIN/B676, http://links.lww.com/PAIN/B677 and http://links.lww.com/PAIN/B678.
